# Close concordance between pulmonary angiography and pathology in a canine model with chronic pulmonary thromboembolism and pathological mechanisms after lung ischemia reperfusion injury

**DOI:** 10.1007/s11239-015-1268-5

**Published:** 2015-08-19

**Authors:** Chaosheng Deng, Dawen Wu, Zhenguo Zhai, Qichang Lin, Zhanghua Zhong, Yuanhua Yang, Qunlin Chen, Ningfang Lian, Shaoyong Gao, Minxia Yang, Kaixiong Liu, Chen Wang

**Affiliations:** Division of Respiratory and Critical Care Medicine, First Affiliated Hospital of Fujian Medical University, Fuzhou, 350005 Fujian China; Division of Respiratory and Critical Care Medicine, Beijing Institution of Respiratory Medicine, Beijing Chaoyang Hospital, Capital Medical University, Beijing, 100020 China; Department of Medical Imaging, First Affiliated Hospital of Fujian Medical University, Fuzhou, 350005 Fujian China; Beijing Key Laboratory of Respiratory and Pulmonary Circulation, Institute of Respiratory Medicine, Beijing Hospital, Ministry of Health, Beijing, 100730 China

**Keywords:** Pulmonary thromboembolism, Pulmonary angiography, Lung ischemia reperfusion injury, Animal model, Pathology

## Abstract

To investigate the pulmonary angiography and pathology in a canine model with chronic pulmonary thromboembolism (PTE). The cylindrical blood clots were selectively introduced into the left (n = 10) or right (n = 20) lower pulmonary arteries of dogs. Pulmonary arteriography (PA) was performed before or after embolization. The values after embolization and baseline of mean pulmonary arterial pressure, pulmonary vascular resistance, cardiac output had changed. After 1 or 2 weeks’ embolization, local PA demonstrated the abrupt cut-off perfusion defects or webs, bands, and abrupt vascular narrowing. 2 weeks after embolization, the pathology showed that the fibrin networks of the thrombi had multiple recanalization channels, and pulmonary artery had the concentric, lamellar (onion-like) intimal hyperplasia, multilayered, irregular arrangements of endothelial cells, and the infiltration of inflammatory cells. After embolectomy-mediated reperfusion, 2 weeks’ subgroup showed destroyed and incomplete alveolar structures, and a large number of exudative cells, primarily neutrophils, and exudate. There close concordance between pulmonary angiography and pathology in a canine model with chronic PTE. The LIRI mechanisms after embolectomy-mediated reperfusion involve the destroyed, incomplete alveolar structures, and infiltration of inflammatory cells, primarily neutrophils.

## Introduction

**Fig. 1:**
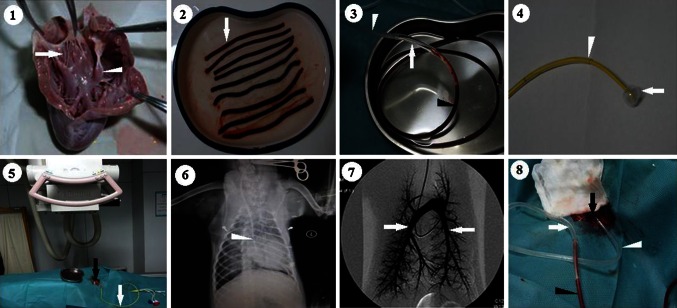
The procedures of establishing the animal model. The dog’s pathological results showed that pulmonary emboli released from peripheral veins can be impeded by the right heart valves, papillary muscle (**1**, *white arrow head*) and chordae tendineae (**1**, *white arrow*). The cylindrical, autologous blood clots were formed by the tube sections (**2**, *white arrow*). After 8 h, the clots were gently aspirated into another PVC tube (tube I) (**3**, *black arrow head*). The tube II (**3**, **4**, white arrow head) was guided by the Swan-Ganz float catheter (**4**, *white arrow*) to float selectively into the left or right lower pulmonary artery under fluoroscopy (**5**, **6**). The tube I and II were connected with another, larger PVC tube (**3**, *white arrow*). Digital subtraction pulmonary angiography (PA) was performed before embolization and showed no filling defects within both pulmonary artery (**7**, *white arrow*). The blood clots prepared inside tube I (**8**, *black arrow head*) were infused into the lower pulmonary artery through tube II (**8**, *white arrow head*) by the dissected right external jugular vein (**8**, *black arrow*)

Pulmonary thromboembolisms (PTEs) are currently the third most common cause of death among hospitalized patients [1]. Animal models of PTE have helped enhance our understanding of the pathogenesis and pathophysiological changes of this syndrome, have suggested methods for diagnosis, and provided a means for evaluating new pharmaceutical-based prophylactic and therapeutic approaches [2]. In the broadest terms, these models can be categorized as being induced by using an injected thrombus or a foreign body. The injected clot model provides a closer representation of PTEs; however, the extent and persistence of the resultant pulmonary vessel occlusion can be difficult to control because of the target animal’s remarkably efficient fibrinolytic system [3, 4]. To counter the effects of the fibrinolytic system, tranexamic acid (TXA)—an inhibitor of plasmin—can be used to inhibit endogenous fibrinolysis in animals [4, 5]. The irregular sizes and volumes of ex vivo-produced clots allow them to flow freely into different pulmonary arteries. Moreover, the pathological results show that pulmonary emboli released from peripheral veins or the vena cava can be impeded by the right heart valves, papillary muscle and chordae tendineae (Fig. 1: 1), as often observed in clinical cases [5, 6]. All these aspects influence the effects of PTE on hemodynamics, cardiac function, and other such parameters [7]. Therefore, experimental models that more accurately reflect the predicted in vivo effects of an intervention would facilitate the assessment and comparison of different antithrombotic agents, and their efficacy and long-term outcomes in PTE treatment [8]. Moreover, lung ischemia reperfusion injury (LIRI) may occur in the region of the affected lung, after thrombolytic therapy, pulmonary embolectomy, or thrombarterectomy in patients with chronic thromboembolic pulmonary hypertension (CTEPH) [9] [10]. The mechanisms of LIRI in PTE are indistinct because of the difficulty in establishing an appropriate ischemia–reperfusion model of PTE.

The canine genome exhibits greater fibrinolytic system homology with the human genome as compared to other mammalian models of thrombotic disease [11, 12]. Interestingly, Virchow’s original description of a canine model focused on the development of animal models of thrombosis that mimicked the human condition [13]. Therefore, in the present study, we aimed to establish a modified canine PTE model involving blood clots that were selectively introduced into the intended specific pulmonary lobar artery and explore the probable pathological and cellular mechanisms of LIRI after the embolectomy.

## Materials and methods

### Animals and groups

The various procedures were approved by the Fujian Medical University Institutional Animal Care and Use committee, and all experiments were conducted in accordance with the Guide for the Care and Use of Laboratory Animals (United States National Institutes of Health, Bethesda, MD). Forty, healthy, 2-year-old dogs (weight, 20 ± 1.9 kg) were divided into 2 groups. The group LL (n = 10) animals had 3-segmented, cylindrical, autologous blood clots introduced into their left main lower pulmonary arteries for 1 week mainly for observing the feasibility of selective embolization. The group RL (n = 30) animals had similar cylindrical blood clots selectively introduced to embolize the right main lower pulmonary arteries for monitoring and comparing vital signs, blood gases, and hemodynamic parameters. The group RL was subdivided into 3 subgroups. The first of these was the Sham subgroup (n = 10). These animals underwent the same procedures as those in the other subgroups, except that 0.9 % NaCl was infused into the lower pulmonary artery of each animal, instead of blood clots, and the animals were observed for 2 weeks. The 1-week subgroup (n = 10) received 3-segmented, cylindrical, autologous blood clots. These clots were introduced into the right lower arteries and the animals were observed for 1 week. The 2-week subgroup (n = 10) underwent the same procedures as the 1-week subgroup, but these animals were observed for 2 weeks. In 5 of the animals in this subgroup, embolectomies were performed for reperfusion, based on the exact location of the thrombus. In the other 5 animals, the lungs were dissected and the lower pulmonary arteries, with the thrombi, were incised for observation.

### Establishing a modified experimental LIRI canine model of PTE

#### Preparation of the cylindrical autologous blood clots

Autologous blood (20 mL) was extracted from the saphenous vein of each dog, using a 20-mL syringe, and rapidly injected into 3-segmented, sterile intravenous polyvinyl chloride (PVC) tubes (Shanghai Muhe Medical Material, Shanghai, China) at room temperature. The tube sections were 7-cm-long, with inner diameters of 4 mm, and were used to form the cylindrical, autologous blood clots. After 8 h, the clots were gently aspirated into another PVC tube (tube I; length, 25 cm, inner diameter, 5 mm) for later use (Fig. 1: 2, 3).

#### Establishing the animal model and pulmonary angiography

Each dog was anesthetized with intravenous propofol (2.5 mg/kg) and intraperitoneal 3 % sodium pentobarbital (0.5 mL/kg; Changzhou Medical Material, Changzhou City, China), and intubated. Thereafter, each animal received a single bolus dose of intravenous TXA (110 mg/kg). Vital signs, including respiratory rate (RR), heart rate (HR), and mean blood pressure (MBP), were recorded according to the experimental scheme. Blood pressure was monitored using a right femoral artery cannula (Changzhou Medical Material). Arterial blood pH, oxygen partial pressure (PaO_2_), and carbon dioxide partial pressure (PaCO_2_) were also periodically monitored. A Swan-Ganz float catheter (Edwards Lifesciences, Irvine, CA, USA) was used to guide a 40-cm PVC tube (inner diameter, 5 mm; tube II) to float selectively into the left or right lower pulmonary artery, under fluoroscopic guidance (Fig. 1: 4). The right external jugular vein was dissected and cannulated with a 7-Fr sheath (Fig. 1: 5) (Shanghai Muhe Medical Material). The Swan-Ganz catheter was connected to a pressure transducer and a multi-channel signal analysis system (Shanghai Alcott Biotech, Shanghai City, China) to monitor the central venous pressure (CVP), mean pulmonary arterial pressure (MPAP), and pulmonary artery wedge pressure (PAWP); measure the cardiac output (CO) using the thermal dilution method; and calculate pulmonary vascular resistance (PVR = [(MPAP − PAWP)/CO] × 80). Digital subtraction pulmonary angiography (PA) (GE Healthcare, Little Chalfont, Buckinghamshire, UK) was performed using Ultravist (300 mg/mL, Seling Pharmaceutical, Guzhou, China, Fig. 1: 6, 7). After guiding tube II into the left or right lower artery, the Swan-Ganz catheter was extracted from inside tube II. Tubes I and II were connected with another, larger PVC tube (length, 3 cm; inner diameter, 6 mm) (Fig. 1: 3), and the blood clots prepared inside tube I were infused into the lower pulmonary artery through tube II, using gentle syringe pressure (Fig. 1: 8). Local PA was performed to confirm the embolism. Thereafter, the animals were allowed to recover from anesthesia and returned to their regular diet with food and water ad libitum. Oral, enteric-coated indomethacin tablets (0.5 mg/kg, 3 times/day for 3 days) were provided for pain relief, and oral TXA (110 mg/kg, every 12 h, for the duration of the experiment) was provided to inhibit endogenous fibrinolysis. Prophylactic penicillin (80,000 U/kg, twice daily for 1 week) was also provided for prevention of infections.

#### Euthanasia and thrombopathology

One or two weeks after embolization, local PA was performed once more and various parameters were recorded. In the reperfusion subgroup, reperfusion (embolectomy) was performed and the animals were mechanically ventilated. Briefly, a right thoracotomy was performed through the fifth intercostal space. The right lower pulmonary lobe was mobilized, after dividing the pulmonary ligament, and the hilar structures were then dissected free. A Fogarty arterial embolectomy was performed as soon as possible, based on the exact location of the thrombus. Thereafter, the lower pulmonary artery was observed for reperfusion changes for 6 h. All animals were euthanized by exsanguination under deep anesthesia. The lungs were removed and the lower pulmonary arteries, with thrombi, were dissected, and fixed in a 10 % aqueous formalin solution.

### Investigating the effects and pathological, cellular mechanisms after chronic PTE and LIRI

A thrombo-pathology study was conducted using histological and paraffin sections stained with hematoxylin and eosin (HE) and phosphotungstic acid-hematoxylin (PTAH). The gross lung appearance were investigated in four groups. The thrombus was shown after embolectomy from the right lower pulmonary lobar artery. The formalin-fixed lung tissues were embedded in paraffin, cut into 4-mm-thick tissue slices and were stained with HE for investigating.

### Statistical analysis

SPSS 11.0 (IBM, Armonk, NY, USA) software was used for the statistical analyses. Numerical parameters with normal Gaussian distribution (according to the Kolmogorov–Smirnov test) were expressed as mean ± standard deviation ($\overline{x}$ ± *s*). Differences in the parameters, among the post-embolization time points within each group or subgroup, were analyzed using repeated measures analysis of variance; a *P* value of < 0.05 was considered significant.

## Results

### Pulmonary angiography

**Fig. 2:**
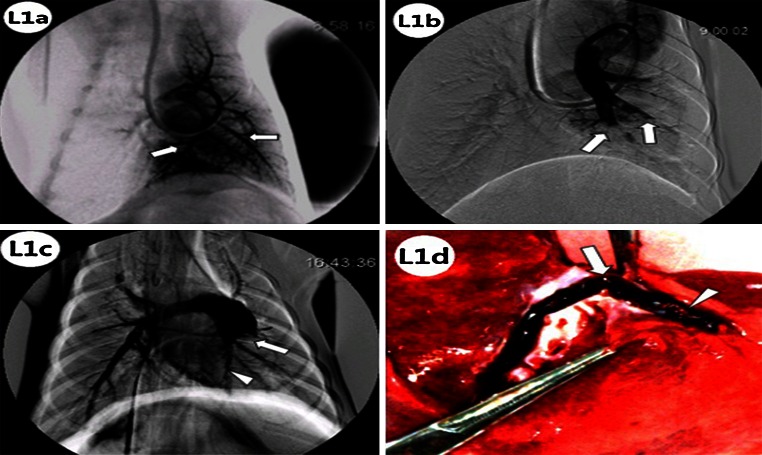
The pulmonary arteriography (PA) and macroscopic pathology of pulmonary artery in Group LL were showed. **L1a** In Group LL, normal filling with contrast media (indicated by *white arrows*) was observed in the left lower lobar artery during local PA, before embolization. **L1b** After embolization, local PA showed a left lower lobar artery irregular ramp and cut-off perfusion defects (*white arrows*). **L1c** After 1 week, PA showed arterial wall irregularities, enlarged proximal sections of left lower pulmonary arteries, and abrupt vessel cut-off perfusion defects (*white arrow*) with stria contrast medium filling along the arterial wall (*white arrow head*). **L1d** One week after embolization, macroscopic pathology examination indicated bifurcal, *reddish-brown* thrombi firmly adherent to the pulmonary artery wall (*white arrows*) along with the presence of multiple, irregular, *pink*, granulation-like protrusions on the thrombus surface (*white arrow head*)

Filling defects were not observed in the full or local PAs in the posterior-anterior projection, before embolization (Figs. 1: 7, 2: L1a, 3: R1a). After embolization, the local PA demonstrated an irregular shape of the lower lobar artery with cup-like and cut-off perfusion defects (Figs. 2: L1b, 3: R1b). After 1 week, PA demonstrated arterial wall irregularities, enlarged proximal parts of the lower artery, and abrupt vessel cut-off perfusion defects, with stria contrast medium filling along the arterial wall, or irregular ramp perfusion defects (Figs. 2: L1c, 3: R1c).

**Fig. 3:**
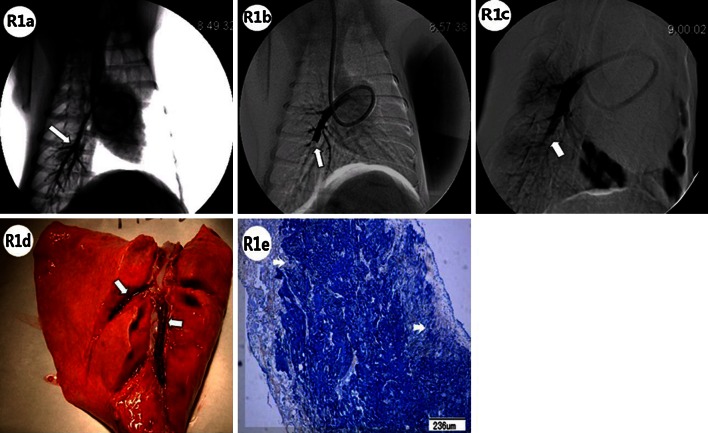
The pulmonary arteriography (PA), macroscopic pathology and pathology of pulmonary artery in the 1-week subgroup of Group RL were showed. **R1a** In the 1-week subgroup of Group RL, good filling with contrast media (indicated by *white arrows*) was observed in the right lower lobar artery on local PA, before embolization. **R1b** After embolization, PA showed right lower lobar artery cup-like perfusion defects (*white arrow*). **R1c** After 1 week, PA showed irregular ramp perfusion defects (*white arrow*). **R1d** 1 week after embolization, the macroscopic pathology revealed bifurcal, *reddish-brown* thrombi, with coarse surfaces, firmly adhered to the right lower lobar artery wall (*white arrows*). **R1e** Pathology showed organized tissue on the surfaces of the thrombi, with *pale orange-pink* covers, and invasive growth (*white arrows*) into the *navy blue* coarse fibrin nests of the thrombus (PTAH stain)

### Parameter changes (Table 1)

#### Vital signs

 Within each PTE group, the RR and HR increased significantly following clot infusion. After embolization, there were no significant changes in MBP, compared with the baseline (*P* > 0.05), among the groups and subgroups.

**Table 1:** Changes in vital signs, blood gases, and hemodynamic parameters among the subgroup ($\overline{x}$ ± s)

Parameters	Group	Subgroup (each n = 10)	Before embolization	After embolization	After 1 or 2 weeks
RR	LL	1-week	23 ± 2	34 ± 2^*^	27 ± 3^*#^
Times/min	RL	Sham	24 ± 3	25 ± 3	24 ± 2
		1-week	23 ± 2	35 ± 3^*^	27 ± 2^*#^
		2-week	24 ± 2	34 ± 3^*^	27 ± 3^*#^
HR	LL	1-week	144 ± 5	185 ± 6^*^	151 ± 5^*#^
Beats/min	RL	Sham	151 ± 5	150 ± 8	149 ± 7
		1-week	143 ± 6	185 ± 5^*^	151 ± 6^*#^
		2-week	152 ± 6	181 ± 7^*^	156 ± 9^*#^
MBP (mmHg)	LL	1-week	108 ± 16	103 ± 17	102 ± 14
	RL	Sham	95 ± 6	96 ± 7	94 ± 11
		1-week	108 ± 18	103 ± 19	102 ± 14
		2-week	110 ± 17	109 ± 15	109 ± 13
pH	LL	1-week	7.32 ± 0.11	7.28 ± 0.10	7.29 ± 0.10
	RL	Sham	7.35 ± 0.05	7.34 ± 0.02	7.32 ± 0.06
		1-week	7.30 ± 0.09	7.28 ± 0.11	7.28 ± 0.09
		2-week	7.32 ± 0.04	7.32 ± 0.06	7.28 ± 0.07
PaO_2_/FiO_2_ (mmHg)	LL	1-week	505.21 ± 56.35	390.21 ± 96.53^*^	497.89 ± 94.23^#^
	RL	Sham	492.89 ± 31.43	488.33 ± 15.35	510.52 ± 41.22
		1-week	507.21 ± 50.21	390.21 ± 93.56^*^	496.99 ± 91.73^#^
		2-week	492.44 ± 48.56	375.88 ± 88.87^*^	467.68 ± 53.21^#^
PaCO_2_ (mmHg)	LL	1-week	38.15 ± 9.69	36.88 ± 5.9	436.69 ± 7.79
	RL	Sham	38.56 ± 3.59	37.33 ± 6.8	336.51 ± 6.88
		1-week	38.66 ± 6.01	37.52 ± 5.1	436.66 ± 6.32
		2-week	36.23 ± 3.99	34.44 ± 3.7	336.89 ± 6.01
MPAP (mmHg)	LL	1-week	15 ± 2	21 ± 3^*^	20 ± 3^*^
	RL	Sham	14 ± 2	15 ± 2	14 ± 2
		1-week	14 ± 2	22 ± 2^*^	20 ± 3^*^
		2-week	14 ± 2	22 ± 3^*^	17 ± 2^#^
CO (L/min)	LL	1-week	3.13 ± 0.32	2.28 ± 0.25^*^	2.89 ± 0.33
	RL	Sham	3.02 ± 0.29	2.93 ± 0.29	2.92 ± 0.29
		1-week	3.01 ± 0.29	2.09 ± 0.35^*^	2.60 ± 0.29
		2-week	3.19 ± 0.28	2.19 ± 0.39^*^	3.16 ± 038^#^
PVR (dyne/s/cm^−5^)	LL	1-week	217.33 ± 38.9	548.20 ± 66.10^*^	419.18 ± 56.2^*^
	RL	Sham	211.0 ± 35.55	220.29 ± 41.22	189.21 ± 42.22
		1-week	203.11 ± 38.1	555.12 ± 61.01^*^	422.01 ± 52.2^*^
		2-week	183.12 ± 44.09	401.22 ± 71.11^*^	302.99 ± 50.02^*^

*RR* respiration rate, *HR* heart rate, *MBP* mean blood pressure, *MPAP* mean pulmonary artery pressure, *PaO*_*2*_ oxygen partial pressure, *FiO*_*2*_ fraction of inspired oxygen, *PaCO*_*2*_ carbon dioxide partial pressure, *CO* cardiac output, *PVR* pulmonary vascular resistance

* *P* < 0.05 for the comparison with parameters recorded before embolization. ^*#*^ *P* < 0.05 for the comparison with parameters recorded after embolization

#### Blood gases

After embolization, the PaO_2_/FiO_2_ decreased significantly after embolization (*P* < 0.05); for example, in the 1-week subgroup of group RL, the post-embolization value was 390.21 ± 96.53 mmHg compared to a baseline value of 507.21 ± 50.21 mmHg (*P* < 0.05). These values increased gradually, toward the baseline value, 1–2 weeks after embolization.

#### Hemodynamic parameters

The MPAP and PVR increased as the CO decreased after embolization (*P* < 0.05). For example, in the 1-week subgroup of group RL, the post-embolization MPAP was 22 ± 2 mmHg, compared to a baseline value of 14 ± 2 mmHg (*P* < 0.05), and the post-embolization PVR was 555.12 ± 61.01 dyne/s/cm^−5^, compared to a baseline value of 203.11 ± 38.1 dyne/s/cm^−5^ (*P* < 0.05), with a post-embolization CO of 2.09 ± 0.35 L/min, compared to a baseline value of 3.01 ± 0.29 L/min (*P* < 0.05). After 1 week, the MPAP was significantly higher (20 ± 3 mmHg) than the baseline value (14 ± 2 mmHg) (*P* < 0.05), whereas the CO demonstrated a decreasing trend with a difference that was not significant compared to baseline (*P* > 0.05). After 1 or 2 weeks, the PVR increased significantly, compared with the baseline value. For example, the PVR in the 1-week subgroup was 422.01 ± 52.2 dyne/s/cm^−5^, compared with a baseline value of 203.11 ± 38.1 dyne/s/cm^−5^ (*P* < 0.05); in the 2-week subgroup, the PVR was 302.99 ± 50.02 dyne/s/cm^−5^, compared with a baseline value of 183.12 ± 44.09 dyne/s/cm^−5^ (*P* < 0.05). There were no significant changes in the CVP and PAWP (*P* > 0.05, data not shown) at different time points among the groups and subgroups.

### Pathological and cellular mechanisms after chronic PTE and LIRI

#### Pulmonary artery angiography is closely concordant with pulmonary artery pathology including thrombo-pathology with cellular proliferation

**Fig. 4:**
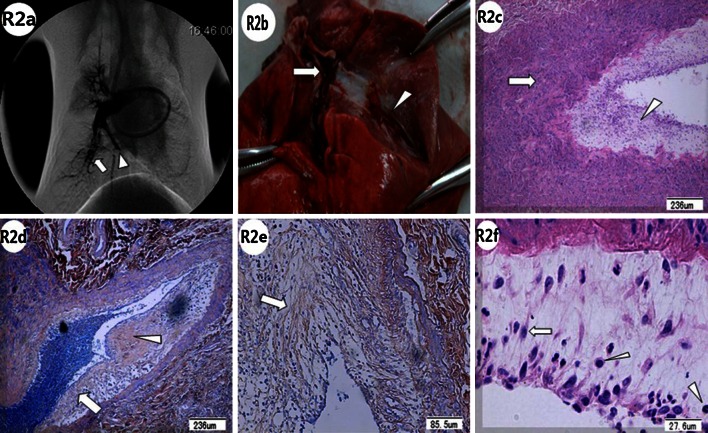
The pulmonary arteriography (PA), macroscopic pathology and pathology of pulmonary artery in the 2-week subgroup of Group RL were showed. **R2a** 2 weeks after embolization in Group RL, local PA showed arterial wall irregularities in addition to webs or bands (indicated by *white arrow*), stria contrast medium filling along the arterial wall, and abrupt vascular narrowing (*white arrow head*). **R2b** Pathology examination revealed a constricted thrombus with an irregular surface, *reddish-brown* proximal region (*white arrow*), and *pink* distal region (*white arrow head*) that was adhered firmly to the pulmonary artery wall. **R2c** Pathology examination showed irregular hyperplasia and hypertrophy of the right lower lobar artery intima (*white arrow head*) and media (*white arrow*) (Hematoxylin and Eosin stain). **R2d** Pathology examination showed irregular vascular walls with neointimal hyperplasia (*white arrow*) and collagen deposition (*white arrow head*) (PTAH stain). There existed a major interspace between intima and media (*black arrow head*). **R2e** Intimal hyperplasia with concentric lamellar (onion-like) structures, composed of some cellular tissue, was separated by elastic fibers (*white arrow*) (PTAH stain). **R2f** Pulmonary arterial neointimal hyperplasia was irregularly arranged with multilayered endothelial cells (*white arrow*) and some inflammatory cell infiltration (*white arrow heads*) (Hematoxylin and Eosin stain)

**Fig. 5:**
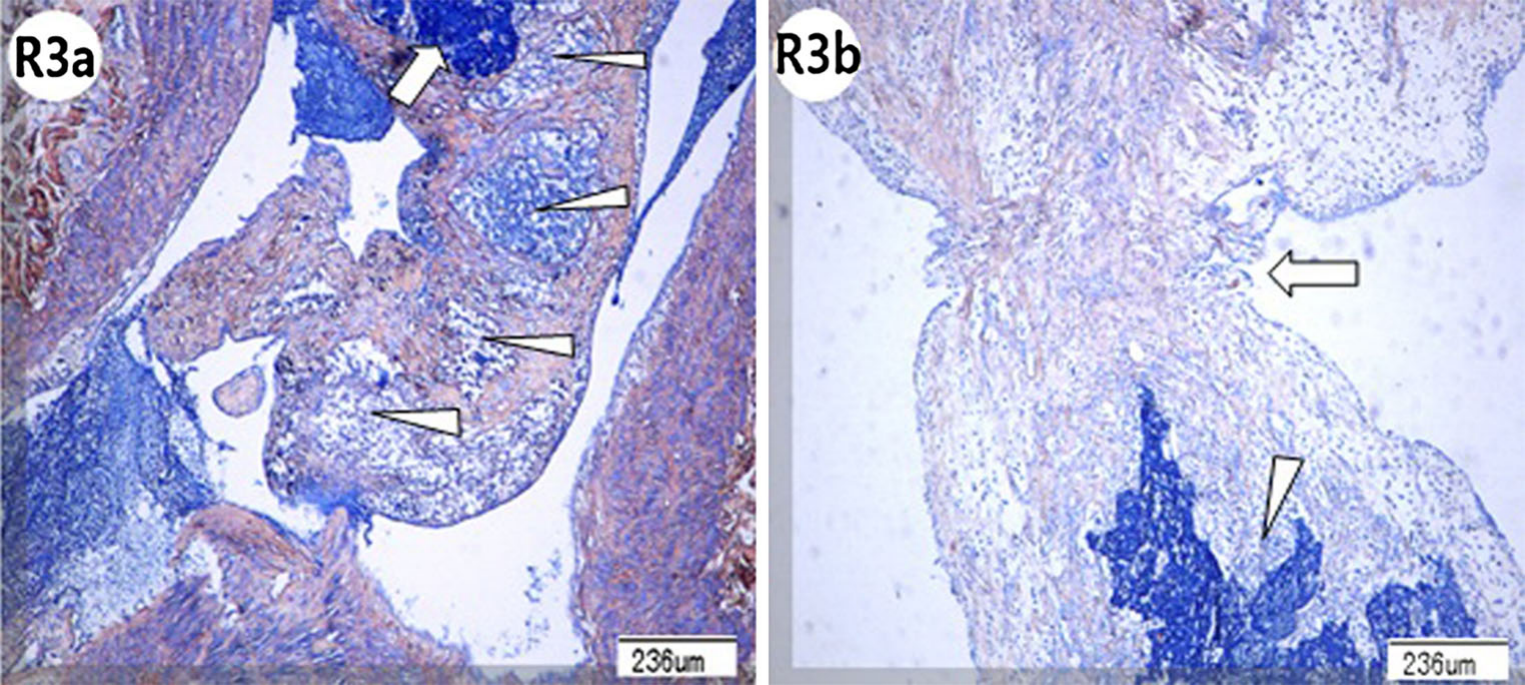
The pathology of thrombi in Group RL was showed 2 weeks after embolization. **R3a** 2 weeks after embolization in Group RL, the thrombi were organized (indicated by *white arrow*) with multiple recanalization channels (white *arrow heads*) (PTAH stain). **R3b** Histological sections indicated that the fibrin networks of the thrombi were invaded (*white arrow head*) by neointimal hyperplasia and collagen from the pulmonary arterial wall (*white arrow*) (PTAH stain)

Pulmonary artery angiography is concordant with the pulmonary artery pathology after the lower pulmonary arteries, with thrombi, were dissected according to the angiography. In the 1-week subgroup of group RL, the pathological examination showed that the organized tissues on the surfaces of the thrombi were pale orange-pink in color with some collagen deposited, and they had invaded the coarse fibrin nests of the thrombus (Fig. 3: R1e). In the 2-week subgroup, there was irregular hyperplasia and hypertrophy of the right lower arterial intima and media (Fig. 4: R2c), irregular vascular walls with neointimal hyperplasia and much more collagen deposition. There may exist an interspace between intima and media (Fig. 4: R2d), and intimal hyperplasia with concentric lamellae (onion-like), composed of some cellular tissue separated by elastic fibers (Fig. 4: R2e). In addition, the pulmonary arterial neointimal hyperplasia was irregularly arranged with multilayer endothelial cells, infiltrated with some inflammatory cells (Fig. 4: R2f). 2 weeks after embolization, in group RL, the thrombi were organized with multiple recanalization channels (Fig. 5: R3a). The histological sections indicated that the fibrin networks of the thrombi were embedded by neointimal hyperplasia from the pulmonary artery wall with collagen deposited (Fig. 5: R3b).

#### Pathological and cellular effects of LIRI after embolectomy

**Fig. 6:**
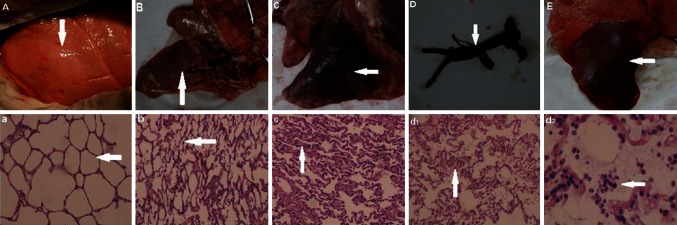
The macroscopic pathology and pathology of pulmonary artery among different groups were showed. The Sham group showed a normal gross lung (**A**, *white arrow*) and normal right lower lung (**a**, Hematoxylin and Eosin stain, 10×, *white arrow*). The 1-week subgroup had *reddish-gray* lungs, with some atelectasis (**B**, *white arrow*) and some collapsed alveolar structures (**b**, Hematoxylin and Eosin stain, 10×, *white arrow*), as well as a few exudative cells in the alveolar space. The 2-week subgroup had dark red lungs, with more obvious atelectasis (**C**, *white arrow*) and more obviously collapsed alveolar structures with thickened alveolar septa (**c**, Hematoxylin and Eosin stain, 10×, *white arrow*). The thrombus is shown after embolectomy from the right lower pulmonary lobar artery (*white arrow*). The thrombus was a complete, elongated strip with multiple branches consistent with the pulmonary artery branches. The reperfusion subgroup had red lungs that were congested and swollen (**E**, *white arrow*), with destroyed and incomplete alveolar structures as well as a large number of exudative cells (*d1*, Hematoxylin and Eosin stain, 10×, *white arrow*), mainly neutrophils (*d2*, Hematoxylin and Eosin stain, 40×, *white arrow*), and exudate

The Sham group showed a normal lung appearance, with pink color (Fig. 6a) and normal intact alveolar structures in the right lower lung (Fig. 6a). The 1-week subgroup had reddish-gray lungs, with some atelectasis (Fig. 6b) and some collapsed alveolar structures (Fig. 6b), as well as a few exudative cells in the alveolar space. The 2-week subgroup had dark red lungs, with more obvious atelectasis (Fig. 6c) and more obviously collapsed alveolar structures with thickened alveolar septa (Fig. 6c). The thrombus was shown after embolectomy from the right lower pulmonary lobar artery (Fig. 6d). The thrombus was a complete, elongated strip with multiple branches consistent with the pulmonary artery branches. The reperfusion subgroup had red lungs that were congested and swollen (Fig. 6e), with destroyed and incomplete alveolar structures as well as a large number of exudative cells (Fig. 6d1), primarily neutrophils, infiltration into the alveolar space and exudate(Fig. 6d2).

## Discussion

### A modified chronic PTE model and pulmonary angiography findings

In most cases, obtaining PTE site samples from human patients is not possible; therefore, animal models are often used for studying the disease.

#### Major hemodynamic changes in the model

In our model, we were able to embolize specific lobar arteries with blood clots. In patients with PTE, the lower pulmonary lobar artery is commonly involved due to its extensive circulation [14]; hence, we aimed to embolize these vessels in the currently described model. After embolization, the MPAP and PVR increased, and then gradually reduced as the thrombi resolved, or circulatory reorganization or recanalization occurred. However, even 2 weeks after embolization, the PVR remained significantly higher than the baseline values, mimicking some hemodynamic changes associated with CTEPH.

#### Pulmonary angiography findings in the model

PA remains the gold standard modality for diagnosing PTE. During 1 or 2 weeks after embolization, PA demonstrated arterial wall irregularities, enlarged proximal portions of the lower pulmonary artery, abrupt vessel cut-off perfusion defects, stria contrast medium filling along the stiff arterial walls, and abrupt vascular narrowing, which are common manifestations of chronic PTE or CTEPH.

A dog’s life span is generally 11–14 years, which is approximately one-sixth of the human lifespan. Thus, we speculated that the pathophysiological changes occurring within 2 weeks, in a dog, may somewhat reflect the changes observed over 12 weeks in humans, which is the chronic PTE or CTEPH threshold time [15–17]. Chronic PTE are usually recognized as being synonymous with CTEPH. However, serial pulmonary angiographic and lung scan studies have revealed that approximately 15–25 % of acute PTE patients show only partial resolution of their pulmonary vascular obstructions in follow-up lung scans, performed 3–4 months after the primary embolic event [18–20]. Over a period of months or years, approximately 3 % of patients with acute PTE develop CTEPH [21]. The underlying pathophysiological mechanisms are largely unknown [22] [23]. Many studies have shown that some patients demonstrate “chronic pulmonary embolism” features, without evidence of chronic pulmonary hypertension, however, they may develop pulmonary hypertension [24–26]. Thus, this new insight has added additional caveats about the natural history of acute pulmonary embolisms that should be considered [22] [27]. In our study, 2 weeks after embolization, the appearance of webs or bands and abrupt vascular narrowing and intimal irregularities was consistent with the pulmonary angiography findings of CTEPH cases [28–30] and may represent a multiply recanalized thrombus [31].

#### Close concordance between pulmonary artery angiography and pulmonary artery pathology in the model

Pulmonary artery angiography is closely concordant with the pulmonary artery pathology after the lower pulmonary arteries, with thrombi, were dissected according to the angiography in our model. Therefore, a modified and reproducible canine PTE model can be established by selectively introducing blood clots into specific pulmonary lobar arteries using the Swan-Ganz catheters under fluoroscopic guidance.

### Effects and pathological, cellular mechanisms after chronic PTE and LIRI

#### Pulmonary vascular wall remodeling mechanisms after chronic PTE in the model

As mentioned above, the macroscopic pathology examination demonstrated that the thrombi consistent with the filling defects shown on PA. In histological sections of our model, the medial or neointimal hyperplasia, as well as the invasion of collagen into the thrombus fibrin networks, demonstrated the progress and severity of pulmonary vascular wall remodeling, which is associated with the duration of embolism,as shown in the endarterectomized tissues from CTEPH cases [32]. Multiple recanalization channels within the organized thrombus imply the predominance of fibrinolytic activity and the number of recanalized lesions correlates with the reduction in PVR [31].

#### Cellular and molecular mechanisms after chronic PTE in the model

In the 2-week subgroup in our model, many findings demonstrated the cellular and molecular mechanisms after chronic PTE. These findings included the concentric, lamellar (onion-like) intimal hyperplasia, with fibrous septa; multilayered, irregular arrangements of endothelial cells; and the infiltration of inflammatory cells. These findings can also be reflected by the histological examinations of endarterectomized tissues from patients with CTEPH [14, 32, 33]. The microenvironment provided by the unresolved clot and inflammatory cells may stimulate erroneous cell proliferation, promote the endothelial-mesenchymal transition, cause endothelial injury and/or induce endothelial cell (EC) dysfunction [33, 34]; furthermore, the infiltration of inflammatory cells, such as leukocytes or monocytes, into the vascular wall may release proinflammatory cytokine macrophage chemoattractant protein-1 (MCP-1) and interleukins 1 and 6, which can potentially act as chemoattractants for fibroblasts or smooth muscle cells [15]. Because of ischemia, some collapsed alveolar structures, thickened alveolar septa, and collagen fibers stained blue and a few exudative cells, in the alveolar space were also demonstrated in our recent study [35]. All these are critical for future investigations into the of the disease and for the development of novel and therapeutic approaches.

#### The mechanisms of LIRI after embolectomy-mediated reperfusion

According to our experimental model, precise embolization into the intended location also facilitates the use for preclinical investigations into interventional management or open pulmonary thromboembolectomy as clinical practice [36, 37]. In our model, we investigated LIRI after performing the embolectomy to study the exact location of the thrombus and related effects. The experimental and clinical observations suggest that the main dysfunctional characteristic of LIRI is an increase in pulmonary microvascular permeability, and that the transendothelial migration of inflammatory cells may be a critical step in the development of dysfunction after LTX treatment, and may be a source of inflammatory mediators [38]. In our study, the incomplete and destroyed alveolar structures, in conjunction with large numbers of exudative cells, mainly neutrophils, and exudation distal to the clot, after embolectomy in the reperfusion subgroup following 2 weeks of ischemia, provided strong experimental evidence of similar mechanisms for LIRI in PTE and lung transplantation. In addition, the methods and procedures used in our model can also be used to selectively embolize other target organs or blood vessels, such as certain brain vessels, with the guidance of a Swan-Ganz float catheter.

### Limitations and clinical implications

Although the process of chronic PTE was mimicked in our research, some distinctions remain between our model and the actual process. Blood clots induced in vitro or ex vivo are distinct from the laminar and heterogeneous venous thrombi formed within a deep vein [39]. An ideal “PTE animal model” does not exist because animals do not develop spontaneous DVTs [2].

Pulmonary vascular wall remodeling and some cellular and molecular mechanisms after chronic PTE are demonstrated in our model. The possible interspace between proliferative intima and media in our model may prove the possibility for thrombarterectomy, as successfully performed in the patients with CTEPH in clinical pracitce. After reperfusion, the incomplete and destroyed alveolar structure, and cells mainly neutrophils may play an important role in the LIRI in the model, implying anti-inflammation to relieve the LIRI after thrombarterectomy in clinical CTEPH cases. However, there exist difference with the biological processes such as thrombus organiztion between the dogs and human beings. Moreover, the mechanisms of LIRI are profound and may include neutrophil activation, cytokines, ROS, arachidonic acid derivatives, complement, etc., causing cellular damage. Therefore, further studies shoud focus intensively on the interactions among inflammatory response factors during LIRI in PTE. We believe that progress will be made towards an improved understanding of PTEs, including CTEPH, chronic PTE, and LIRI after reperfusion in clinical practice.

A modified canine PTE model can be established by selectively introducing blood clots into specific pulmonary lobar arteries using Swan-Ganz catheters under fluoroscopic guidance. This model with the similar pulmonary angiography findings may mimic the clinical chronic PTE cases. The pathological and cellular mechanisms related to chronic PTE mainly involve the recanalization of thrombi and the remodelling of pulmonary artery with the concentric, lamellar (onion-like) intimal hyperplasia,multilayered, irregular arrangements of endothelial cells, and the infiltration of inflammatory cells. On the other hand, the mechanisms related to the LIRI after embolectomy-mediated reperfusion involve the destroyed, incomplete alveolar structures, and infiltration of inflammatory cells, primarily neutrophils and the detailed mechanisms warrant further investigation.

## Abbreviations

PTEs: Pulmonary thromboembolisms
DVT: Deep vein thrombosis
VTEs: Venous thromboembolisms
PEs: Pulmonary embolisms
TXA: Tranexamic acid
LIRI: Lung ischemia reperfusion injury
CTEPH: Chronic thromboembolic pulmonary hypertension
PVC: Polyvinyl chloride
RR: Respiratory rate
HR: Heart rate
MBP: Mean blood pressure
PaO_2_: Oxygen partial pressure
PaCO_2_: Carbon dioxide partial pressure
CVP: Central venous pressure
MPAP: Mean pulmonary arterial pressure
PAWP: Pulmonary artery wedge pressure
CO: Cardiac output
PVR: Pulmonary vascular resistance
PA: Pulmonary angiography
HE: Hematoxylin and eosin
PTAH: Phosphotungstic acid-hematoxylin
EC: Endothelial cell
MCP-1: Macrophage chemoattractant protein-1
